# Safety and efficacy of a novel fecal microbiota transplantation method using hydrogen nanobubble water without antibiotics or bowel cleansing in children with autism spectrum disorder: an open-label, single-arm study demonstrating improvements in core and comorbidity symptoms

**DOI:** 10.3389/fped.2026.1767346

**Published:** 2026-03-11

**Authors:** Masahiko Shirotani, Shin Shimizu, Kunihiro Kitamura, Yasuhito Mikawa, Reiko Haruna, Yuichi Kawai, Seiji Koide, Ikuko Mohri, Hideo Matsuzaki, Kenji J. Tsuchiya, Yoshimu Tanaka, Taiichi Katayama

**Affiliations:** 1 The Association for Clinical Research of Fecal Microbiota Transplantation Japan, Osaka, Japan; 2 Shirotani Biowellness Clinic, Hyogo, Japan; 3 Shinbiosis Corporation, Osaka, Japan; 4 Kiwakai Kitamura Clinic, Fukuoka, Japan; 5 Natural Art Clinic Yotsuya, Tokyo, Japan; 6 Haruna Clinic, Osaka, Japan; 7 Yuakai Kawai Clinic for Internal Medicine, Osaka, Japan; 8 Koide Clinic, Osaka, Japan; 9 Department of Child Development, Course of Developmental Neuroscience, United Graduate School of Child Development, The University of Osaka, Suita, Osaka, Japan; 10 Research Center for Child Mental Development, University of Fukui, Fukui, Japan; 11 Research Center for Child Mental Development, Hamamatsu University School of Medicine, Shizuoka, Japan; 12 Jinzenkai Tanaka Clinic, Osaka-shi, Osaka, Japan

**Keywords:** anxiety, autism spectrum disorder, depression, fecal microbiota transplantation, gastrointestinal symptoms, gut-brain axis, hydrogen nanobubble, safety

## Abstract

**Background:**

Autism spectrum disorder (ASD) is rising in prevalence, but effective treatments for its core symptoms remain limited. Fecal microbiota transplantation (FMT) has shown promise; however, conventional methods often require antibiotics and bowel cleansing, raising concerns regarding safety and sustainability. We developed a novel FMT method using hydrogen nanobubble water and investigated its efficacy and safety.

**Methods:**

This prospective, single-arm, before-and-after comparative study enrolled 30 children aged 5–12 years with ASD, selected according to inclusion and exclusion criteria. SHIN-1, a Good Manufacturing Practice (GMP)-grade prepared fecal microbial solution from a healthy screened donor, was suspended in hydrogen nanobubble water and administered via enema. Primary outcome was the Social Responsiveness Scale-2 (SRS-2), with objectivity confirmed using Gazefinder as an eye-tracking system. Secondary outcomes included sensory profile [Short Sensory Profile (SSP)], gastrointestinal symptoms [Gastrointestinal Symptom Rating Scale [GSRS], Bristol Stool Form Scale [BSFS]] and Patient Health Questionnaire-4 items (PHQ-4). Statistical analyses employed paired t-tests or Wilcoxon signed-rank tests (*α* = 0.05).

**Results:**

At 30 weeks, fecal microbiota reconstitution was observed, with increases in short-chain fatty acid–producing and typically taxa abundant in developing children. SRS-2 scores decreased 29% (*p* < 0.001), sustained at one year. The classification is as follows; 19 severe cases improved to mild and 6 to normal. Improvements were greater in children without gastrointestinal disorders (45% vs. 24%). Social Communication and Interaction (SCI), Restricted Interests and Repetitive Behavior (RRB), and subscales improved uniformly; sensory, gastrointestinal, and emotional symptoms improved by 30%–61%. No adverse events occurred.

**Conclusion:**

This novel hydrogen nanobubble water–based FMT method was safe and effective, reducing both core and peripheral symptoms of ASD and suggesting broad benefits via the gut microbiota–brain axis.

**Clinical Trial Registration:** https://jrct.mhlw.go.jp/en-latest-detail/jRCTs031230041.

## Graphical Abstract

**Figure:**
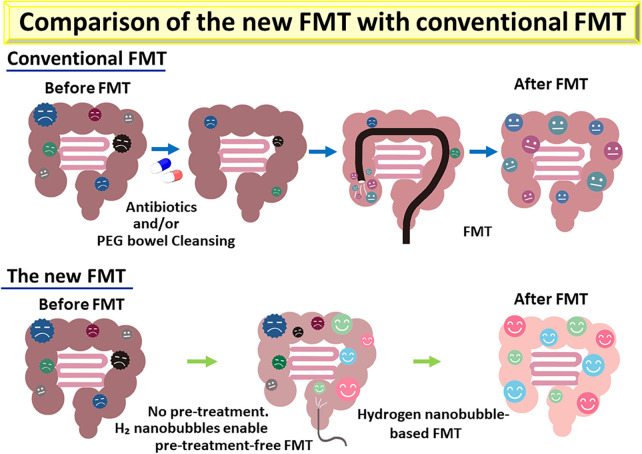

## 1 Introduction

Autism spectrum disorder (ASD) is a neurodevelopmental disorder characterized by core symptoms such as persistent impairments in social communication and interaction, restricted interests and repetitive behaviors, and sensory specificity (1–3). The incidence of ASD is on the rise, posing a significant social challenge. According to a 2023 report from the US Centers for Disease Control and Prevention (CDC), the incidence of ASD among 8-year-olds in 2020 was 1 in 36, a doubling over the past decade (4). However, effective treatments for ASD have yet to be developed. Methods for alleviating the core symptoms of ASD and the resulting difficulties include behavioral therapy and some pharmacological treatments. The former, including various behavioral therapies such as applied behavior analysis, Social Skills Training (SST), and parent training, as well as environmental adjustments, have been proven effective (5, 6). However, behavioral therapy requires detailed behavioral assessment, which is highly individualized and requires the skills and expertise of the assessors, making quality assurance a challenge. Meanwhile, pharmacological treatments such as atypical antipsychotics, selective serotonin reuptake inhibitors, and anti-anxiety drugs are also used, but these are only effective on peripheral symptoms and not on core symptoms (7, 8).

As such, there is no effective treatment that consistently provides stable therapeutic effects for all ASD patients, and the development of new treatments is desired.

The mechanism of ASD pathogenesis has yet to be elucidated, but genetic and environmental factors are thought to play important roles. Recent studies have argued that the composition of the gut microbiota plays an important role in the pathogenesis of ASD and may affect brain function via the gut microbiota-gut-brain axis, which includes the neuroendocrine, neuroimmune, and autonomic nervous systems (9, 10).

Xu et al. analyzed 16S rRNA datasets of gut microbiota from over 1,000 children, including those with ASD and typically developing children, and reported that although there was no significant difference in the alpha diversity of the gut microbiota between the ASD and typically developing groups, there was a significant difference in the composition of the gut microbiota (11). Furthermore, numerous mouse studies have reported a variety of results indicating that the composition of the gut microbiota significantly affects core symptoms of ASD (12, 13).

Against the backdrop of these research trends, there has been intense research into the development of ASD treatments that involve intervention with the gut microbiota.

Although numerous clinical studies on prebiotics and probiotics have been conducted, none have demonstrated clear and reproducible therapeutic effects (14, 15). On the other hand, a vancomycin intervention trial in a small number of children with ASD reported significant improvement in communication skills and some behavioral abnormalities, among ASD characteristics (16). However, this effect was not sustained, and it disappeared within a few weeks due to the formation of Clostridium spores (16).

In parallel with these studies, methodologies are being investigated for treating ASD through gut microbiota transplantation (FMT), which reconstructs the composition of the gut microbiota and modulates the brain-gut axis (16).

Kang et al. administered the antibiotic vancomycin for two consecutive weeks to children with ASD, followed by bowel cleansing with polyethylene glycol (PEG), and then performed FMT (2 days of enema and 50 days of oral administration) (17).

The results of this study showed that 2 years after FMT, 47% of children with severe ASD had progressed to the intermediate, mild, or normal group as assessed by Social Responsiveness Scale (SRS).

However, this method requires continuous administration of vancomycin before FMT (17), which often leads to adverse events. Furthermore, bowel cleansing with PEGand long-term continuous administration of proton pump inhibitors during FMT are also required, as well as the administration of large amounts of donor bacterial flora, making these processes extremely demanding for children with ASD.

On the other hand, Li et al. performed FMT on children with ASD without vancomycin (although the amount of fecal bacterial flora administered was approximately 100 times higher than in Kang et al.'s study). They performed FMT via enema or oral administration after PEG bowel cleansing. Although the severity of ASD was reduced after the treatment, the effect was not sustained and significantly diminished over time (18).

Furthermore, Wu et al. performed a relatively patient-friendly method in which bowel preparation with oral rifaximin and polyethylene glycol was performed daily starting three days before FMT, followed by six consecutive days of FMT. They found that CARS scores at three months post-treatment were reduced by 14.6% compared to baseline, and coexisting disorders such as gastrointestinal symptoms were also reduced (19).

These results demonstrate that it is ultimately difficult to complete the FMT process without administering antibiotics such as vancomycin or rifaximin. Therefore, there is a need for a patient-friendly FMT method that does not require antibiotics or bowel cleansing agents and can be performed with a small dosage (bacterial count). Recently, Shimizu et al. developed a new FMT method using a fecal microbiota solution prepared from healthy stool and hydrogen nanobubble water. This method does not require the administration of antibiotics and allows FMT to be performed with a very low dose of solution (20, 21). Therefore, we investigated the efficacy and safety of this new FMT method for children with ASD.

## 2 Materials and methods

### 2.1 Study design

#### 2.1.1 Study design and ethics

**Figure 1:**
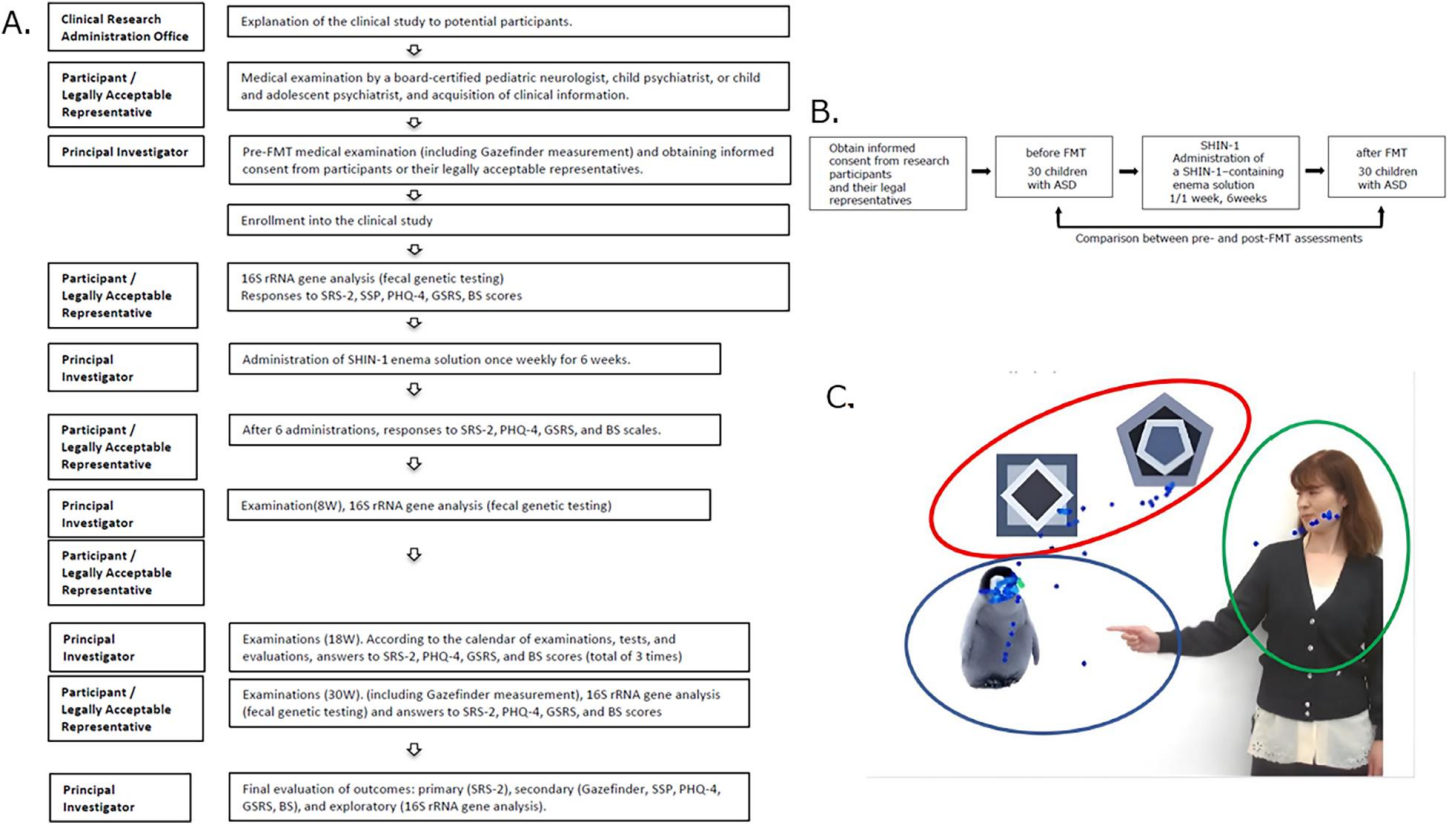
Study protocol and design, evaluation images (Gazefinder). **(A)** An outline of the study is shown below. The left column lists the implementers and departments involved, and the right column lists the details of the study. In this clinical study, all of the implementation items were completed without delay. **(B)** The experimental design of this study is shown in Figure B. This clinical study was conducted as a before-and-after FMT. **(C)** Images used in Gazefinder. This study showed that there was a correlation between the time spent looking at “people and geometric patterns” in Gazefinder's “pointing image” and the severity of SCI (social communication and interpersonal interaction), a major item of the SRS-2, thereby demonstrating that the SCI results obtained in this clinical study are objective.

This study was conducted as a prospective single-arm, before-and-after comparative study, following the steps shown in Figures 1A,B.

The study was reviewed and approved by the Certified Review Board, Hattori Clinic (CRB3180027). All procedures adhered to the Declaration of Helsinki. The trial was registered in the Japan Registry of Clinical Trials with the number jRCTs031230041 before the first participant was enrolled.

#### 2.1.2 Subject selection

##### 2.1.2.1 Inclusion criteria

1. Children between the ages of 5 and 12 who have been diagnosed with autism spectrum disorder (ASD) by a pediatric neurologist, a board-certified psychiatrist, a board-certified child and adolescent psychiatrist, or a board-certified child psychiatrist. (Certificate of diagnosis required; regardless of gender).
2. Subjects in generally good physical health except for gastrointestinal symptoms (as determined by the principal investigator).
3. Subjects who can provide written or, if necessary, illustrated or verbal consent to participate in this study.
4. Subjects who can obtain written consent to participate in this study from a legal representative.

##### 2.1.2.2 Exclusion criteria

1. Subjects currently undergoing pharmaceutical treatment.
2. Subjects who have received antibiotics (excluding topical antibiotics) within the past three months.
3. Subjects who have undergone fecal microbiota transplantation within the past 12 months.
4. Subjects with single-gene disorders exhibiting ASD-like symptoms (such as Fragile X syndrome, Rett syndrome, and Joubert syndrome, which may affect the assessment of efficacy).
5. Subjects with neurosurgery or neurological disorders.
6. Subjects receiving tube feeding.
7. Subjects who are underweight/malnourished (less than 65% of ideal body weight).
8. Subjects who have undergone surgery within the past year or are scheduled for surgery within the next six months.
9. Subjects currently participating in other clinical trials.
10. Subjects deemed inappropriate for study participation by the principal investigator for other reasons.

##### 2.1.2.3 Demographic characteristics of study subjects

The age, sex, height, weight, BMI, and ASD severity of the study subjects are summarized in Table 1.

**Table 1:** Demographic characteristics of study participants.

Category	Subcategory	ASD Children (*n* = 30)
Gender	Female (*n*)	8
	Male (*n*)	22
Age (years, mean ± S.E.M.)		8.07 ± 0.42
	5–6 years old (*n*)	10
	7–8 years old (*n*)	8
	9–10 years old (*n*)	7
	11–12 years old (*n*)	5
BMI (mean ± S.E.M.)		15.90 ± 0.37
Autism severity, *n* (%)^a^	Normal	1 (3.3)^b^
	Mild	1 (3.3)
	Moderate	2 (6.7)
	Severe	26 (86.7)

a In accordance with the inclusion criteria, study subjects diagnosed with ASD by a pediatric neurologist or other specialist were classified using the SRS-2.

b Of the 30 study subjects, one had a normal range in the SRS-2 test.

The male to female study subject ratio in this clinical study was approximately 3:1.

Meanwhile, a recent large-scale study by Shinoyama et al. at Shinshu University found that of the approximately 6 million children born in Japan between 2009 and 2019, approximately 210,000 were diagnosed with ASD, with boys accounting for 76.1% and girls for 23.9%, respectively (22).

This indicates that the gender ratio of study participants in this clinical study was within the national average.

One of the 30 study participants was diagnosed with ASD by a pediatric neurologist, but his SRS-2 raw score total was 42 points (T-score: 56 points), which was within the normal range. As described in detail in the following chapter, this score decreased to 27 points (T-score: 48 points) 24 weeks after administration.

#### 2.1.3 Informed consent

The surrogate mothers of the study participants were given a written explanation of the study and their consent was obtained. The subjects themselves were also given written, diagrammatic, or oral explanations as needed and their consent was obtained.

#### 2.1.4 Preparation of SHIN-1

All steps involved in the preparation of SHIN-1 stock solution described below were carried out in accordance with the Good Manufacturing Practice (GMP) guidelines for investigational drugs.

##### 2.1.4.1 Donor selection and donor stool storage

Six donors were selected and examined by the investigator through various tests and interviews every two months. Simultaneously, the bacterial composition of each donor's stool was evaluated using 16S rRNA gene analysis. As a result, one donor with stable health status and the most stable bacterial composition in their stool was selected (the results of various tests and gene analysis are shown in Supplementary Material 2). This clinical trial was conducted using the stool of the selected donor.

##### 2.1.4.2 Donor stool collection and storage

The collected donor stool was subjected to various tests (Supplementary Data), including 16S rRNA gene analysis, and then stored at −80 °C. Before use, the temperature was gradually lowered to 2–8 °C over 24 h, and SHIN-1 was prepared.

##### 2.1.4.3 Hydrogen nanobubble water

Hydrogen nanobubble water was obtained from Shinbiosis, Inc. The bubbles contained in this water were larger than 200 nm, with a peak at around 450 nm. The bubble count ranged from 2.0 × 10⁹ cells/mL to 7.0 × 10⁹ cells/mL.

##### 2.1.4.4 Preparation, quality, and storage of SHIN-1 stock solution

At room temperature (20 ± 3 °C), approximately 100 g of feces was added to 250 g of hydrogen nanobubble water and stirred with a magnetic stirrer (stirrer length: 3 cm) for approximately 90 min to prepare a suspension. This suspension was first filtered through two layers of sterile gauze, then through ten layers of sterile gauze, and then through another ten layers of sterile gauze to obtain 100–150 g of SHIN-1 stock solution. The stock solution was diluted 1,000-fold to achieve a bacterial concentration of 100–300 cells/μL for transplantation. Bacterial counts were measured using a disposable cell counting chamber. The resulting SHIN-1 stock solution was stored refrigerated (2–8 °C) and remained stable for at least 3 months under these conditions.

##### 2.1.4.5 Preparation and administration of SHIN-1 injection solutions

Intravenous solutions were prepared by adding saline to the SHIN-1 stock solution and mixing each solution with 100 g of saline. Six different intravenous solutions were prepared by mixing 3 g, 5 g, 7 g, 9 g, 11 g, and 13 g of SHIN-1 stock solution with 100 g of saline. The bacterial concentrations in the SHIN-1 stock solution ranged from 1 × 10⁸ to 3 × 10⁸ cells/mL. To minimize subject discomfort, a thin, rubberized 6.5 mm diameter intravenous tube was used for administration. Subject resistance to this administration method was minimal.

#### 2.1.5 Evaluation method

To evaluate the efficacy of the novel FMT on ASD symptoms, the following evaluation systems (a) to (e) were used, centered on the SRS-2. Specifically, core symptoms of ASD were assessed in terms of SCI (social communication and interaction) and RRB (restricted interests and repetitive behaviors). The sensory processing disorder aspect of RRB was assessed using the Sensory Profile-Short Form (SSP). The SCI subscales, social consciousness (Awr), social cognition (Cog), social communication (Com), and social motivation (Mot), were also measured simultaneously.

##### 2.1.5.1 SRS-2

The SRS-2 consists of SCI and RRB scales based on the DSM-5 ASD diagnostic criteria. The SRS-2 crude total score is used to evaluate the effectiveness of interventions in reducing ASD severity. Furthermore, the SRS-2 subitem crude total score evaluates the degree of severity reduction for each subitem. This approach allows for the evaluation of the extent and effect size of the effectiveness of the new FMT method.ASD severity was assessed using the SRS-2 crude total score, with the following criteria: severe (>76), moderate (66–75), mild (60–65), and normal (<59). The change in the number of patients classified by severity level before and after FMT was expressed as a total T-score adjusted for gender and age.

Currently, the Autism Diagnostic Observation Schedule (ADOS) and the Autism Diagnostic Interview-Revised (ADI-R) are recognized as the gold standard for diagnosing ASD and are also used to assess the severity of ASD in children. However, these assessments are conducted by rigorously trained, licensed professionals in collaboration with parents. Therefore, the number of such professionals is limited, and the time required for behavioral observation and questioning places a significant burden on both parents and children.

In contrast, the SRS-2, which is completed by parents and scored by a human examiner, overcomes these challenges and is thought to provide a simple and accurate assessment of the effectiveness of the new FMT method.

##### 2.1.5.2 Gazefinder

As mentioned above, the SRS-2, used as the primary endpoint, has been reported to demonstrate high diagnostic accuracy without introducing bias (23, 24). To ensure that the SRS-2 results were reliable and unbiased, this study evaluated the results using the Gazefinder, a well-known objective test for ASD, as a control. The diagnostic ability of the Gazefinder for ASD has already been extensively studied (25, 26). Fujioka et al. reported that the Gazefinder can assess the “social deficit” of ASD based on attention patterns to “social information” specific to ASD and demonstrated high diagnostic accuracy in differentiating children with ASD (27, 28). In this study, we demonstrated that the duration of gaze on people and geometric patterns in the Gazefinder prompt images (Figure 1C) used by Fujioka et al. correlated with the severity of SCI, a key component of the SRS-2, supporting the objectivity of the SRS-2 results (27).

##### 2.1.5.3 Short sensory profile (SSP)

The effectiveness of the new FMT for hypersensitivity, hyposensitivity, and hyporesponsiveness/sensation seeking was evaluated before and after FMT by SSP.

##### 2.1.5.4 GSRS and bristol stool form scale (BSFS)

The effectiveness of the new FMT method for gastrointestinal disorders was evaluated in subjects with gastrointestinal abnormalities observed using the GSRS (Gastrointestinal Symptom Rating Scale). The BSFS was used to evaluate changes in constipation and diarrhea in 30 subjects based on the change in the BS score before and after FMT.

##### 2.1.5.5 Patient health questionnaire-4 items (PHQ-4)

The PHQ-4 and its subitems, PHQ-2 and Generalized Anxiety Disorder-2 (GAD-2), were used to evaluate the effectiveness of the new FMT method in reducing depression and anxiety in subjects with depression or anxiety symptoms (29).

##### 2.1.5.6 Statistical analysis

Statistical analysis for this study was performed using MedCalc software (version 22.023, MedCalc Software Ltd, Belgium) as follows: To test for differences in clinical parameters (e.g., SRS-2) before and after transplantation, a paired t-test was used if the difference (change) followed a normal distribution. If the difference did not follow a normal distribution, a Wilcoxon signed-rank test was used. The significance level (*P* value) was set at 5%, and a *P* value of less than 0.05 was considered to indicate a statistically significant difference between clinical parameters before and after transplantation. Normality was assessed using the Shapiro–Wilk test.

## 3 Results

### 3.1 Reconstruction of the gut microbiota with a new FMT method

Unlike previous studies (30–33), the new FMT method does not use antibiotics or intestinal cleansing agents, but instead uses very low doses of drugs to reconstruct and maintain the gut microbiota in children with autism spectrum disorder (ASD).

**Figure 2:**
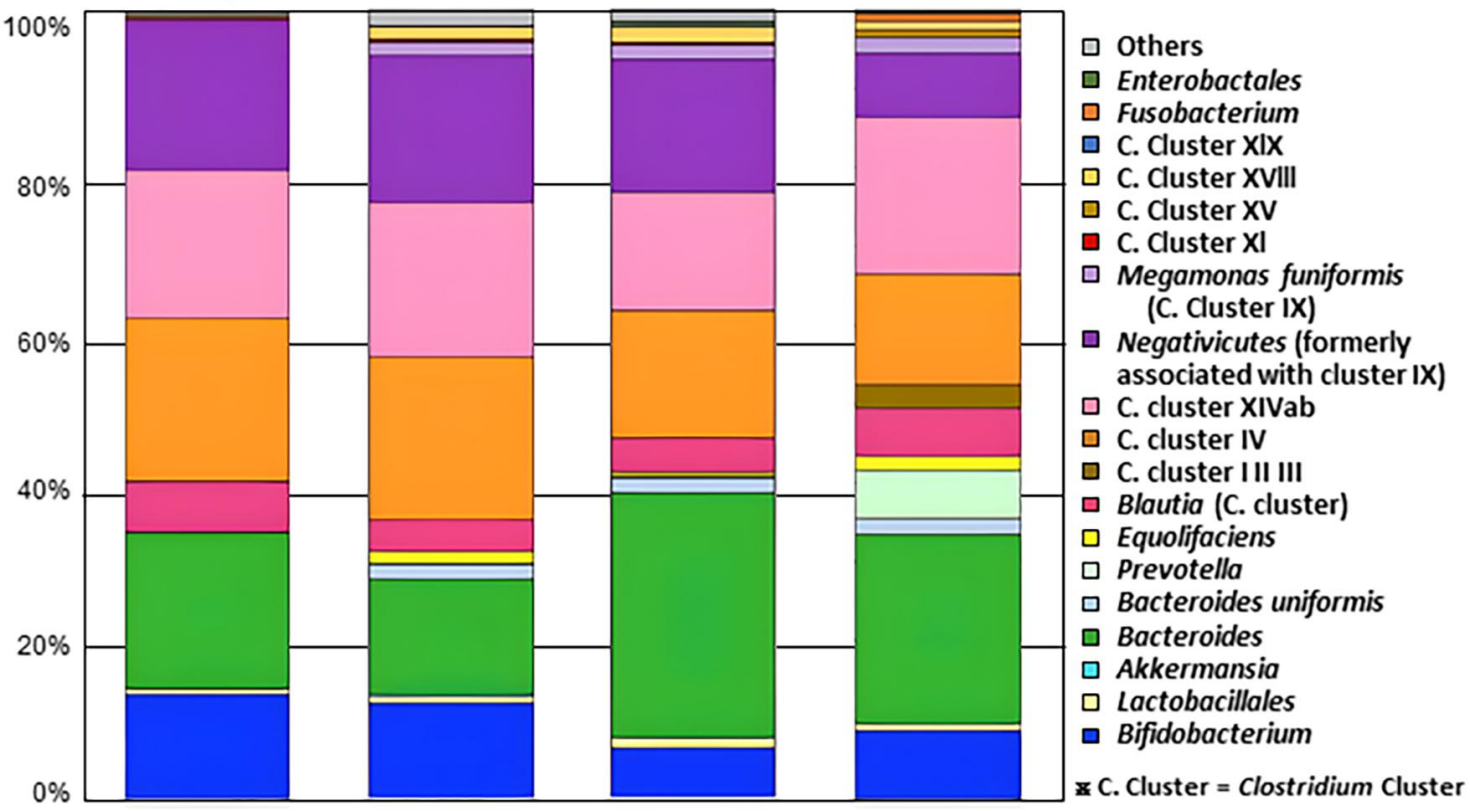
Changes in the gut microbiota composition in children with ASD before and after SHIN-1 administration. Data shows an example of the results of 16S rRNA gene analysis of the gut microbiota composition in donors and subjects before and after FMT (SHIN-1 treatment). 24 weeks after the end of treatment. SHIN-1 was administered once a week from week 1 to week 6, for a total of 6 doses.

Figure 2 shows an example of the results of 16S rRNA gene analysis of the gut microbiota composition in donors and subjects before and after transplantation. 24 weeks after the end of treatment, there was an increase in *Bacteroides* which produce short-chain fatty acids that have a significant impact on the gut environment and immune system. The levels of *Bacteroides* which were low before treatment, increased. Furthermore, there was an increase in the levels of *Clostridium.Cluster* (C. Cluster) XVIII, which is rare in children with ASD but abundant in typically developing children. There was also an increase in *Prevotella, Akkermansia, Fusobacterium, and Bacteroides uniformis*, which are less common in children with ASD but more common in typically developing children (34–38) (Figure 2).

### 3.2 Efficacy of the new FMT

#### 3.2.1 SRS-2

**Figure 3:**
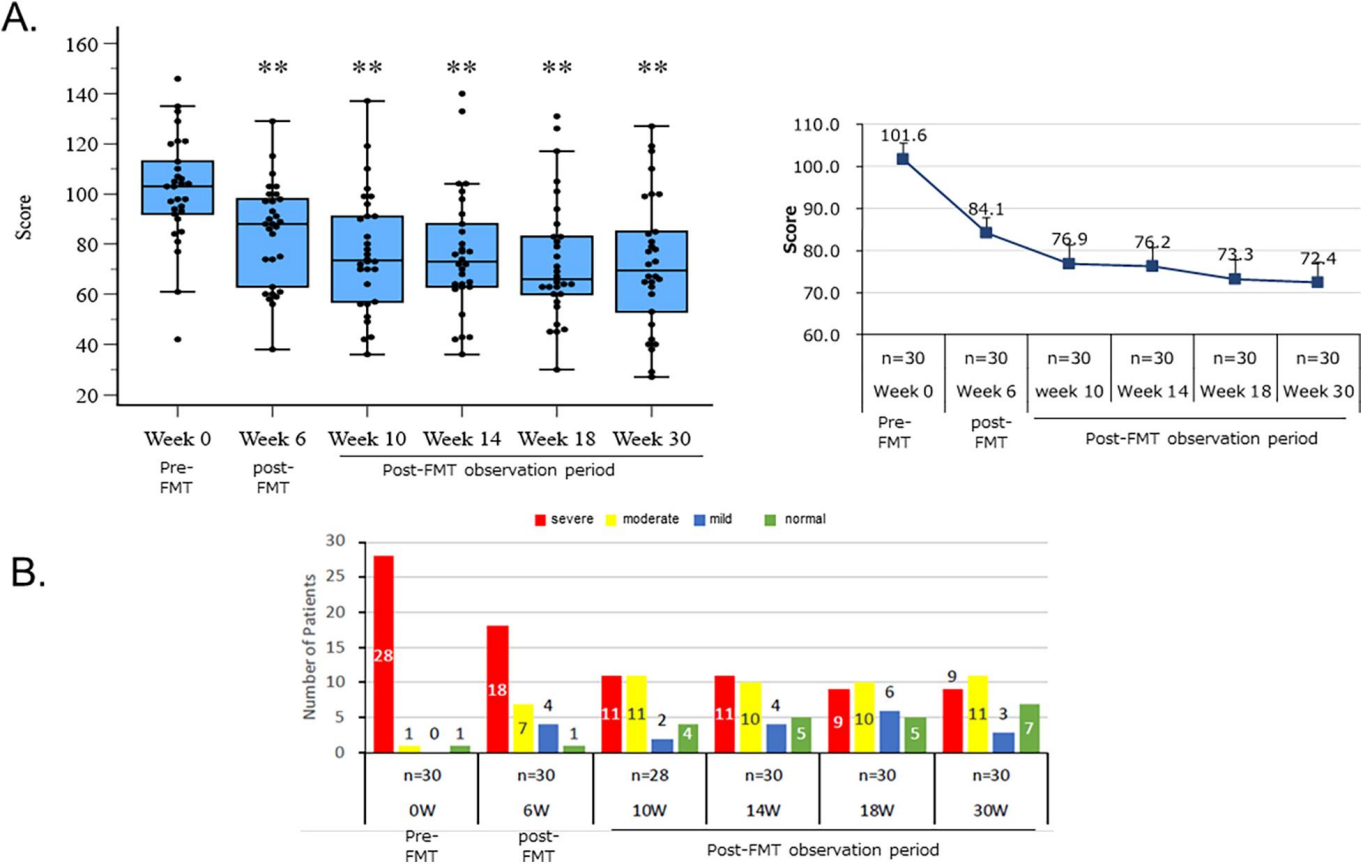
The effect of the new FMT method on reducing the severity of ASD. **(A)** The effect of a new FMT method on reducing the severity of ASD using the SRS-2 total raw score. Box-and-whisker plots of SRS-2 total raw scores at baseline (Pre) and 30 weeks after SHIN-1 intervention (Post). ***p < 0.001, paired t-test or Wilcoxon signed rank test. **(B)** Change in the number of patients by severity before and after FMT. Each column represents the number of patients. Severity was classified by the SRS-2 total T score, and indicated as red: severe, yellow: moderate, blue: mild, and green: normal.

The effect of the new FMT method on reducing ASD severity was assessed using the SRS-2 before and at 6, 10, 14, 18, and 30 weeks after the start of FMT. The results, shown in Figure 3A, demonstrate that the new FMT method significantly reduced symptoms in children with ASD. Specifically, ASD severity in 30 subjects was reduced by approximately 29% compared to before the start of FMT (*p* < 0.001).

Furthermore, Figure 3B shows the change in the number of patients classified by severity between before and 30 weeks after FMT administration. Severity was measured using the SRS-2 total T-score, adjusted for gender and age.

Before FMT, 28 of the 30 study participants had a severe condition, 1 had a moderate condition, and 1 had a condition within the normal range. Thirty weeks after FMT, 19 of the 28 patients (approximately 68%) with a severe condition and 1 with a moderate condition progressed to a mild condition, for a total of 20 patients (approximately 69%), with 6 of these progressing to the normal range. These results demonstrate that the new FMT method is significantly effective in reducing the severity of ASD (Figure 3B).

In the previous study by Kang et al. and Li et al., all subjects had gastrointestinal disorders (ages 30–33). In contrast, this clinical trial included 22 patients with gastrointestinal disorders and 8 patients without gastrointestinal disorders. The results of this clinical trial were analyzed based on the presence or absence of gastrointestinal disorders.

In the 22 participants with gastrointestinal disorders, ASD severity decreased by 24% (*p* = 0.001), while in the 22 participants without gastrointestinal disorders, severity decreased by 45% (*p* < 0.001), with the mean SRS-2 score moving into the normal range (data not shown). These results are interesting compared with previous reports. Further study is needed to confirm these findings.

##### 3.2.1.1 SRS-2 subscales (SCI and RRB)

**Figure 4:**
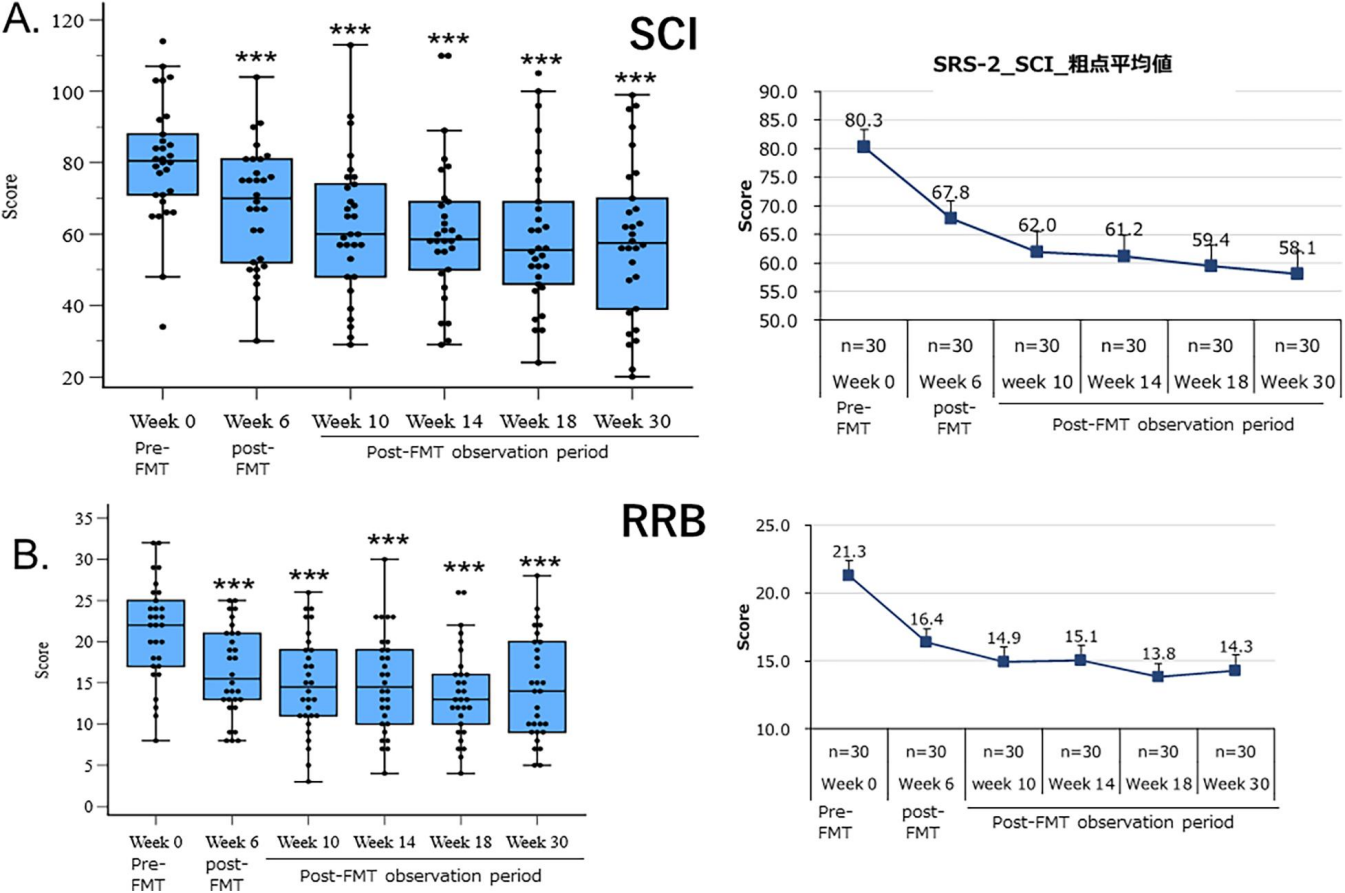
The effect of the new FMT method on reducing the severity of SRS-2 sub-category. Upper: SCI. Lower: RRB. (Left) Boxplot of SRS-2 SCI. Paired *t*-test or Wilcoxon signed-rank test (Post-FMT vs. Pre-FMT); ***p < 0.001. (Right) Mean SCI progression. Each data point represents the mean ± S.E.M. of *n* = 30.

Figures 4A,B shows the results of the SCI and RRB assessments consistent with the DSM-5 ASD diagnostic criteria. The mean total SCI score for the 30 participants decreased from 80.3 before FMT to 58.1 30 weeks later. This represents a 28% (*p* < 0.001) decrease, comparable to the overall decrease in ASD severity shown in Figure 3.

We also examined the effect of the new FMT method on RRB severity. According to the results shown in Figure 4B, the mean RRB severity score for the 30 participants decreased from 21.3 before FMT to 14.3 after 30 weeks. This corresponds to a reduction of approximately 33% (*p* < 0.001). Notably, the reduction in RRB severity was similar to the reductions observed in ASD severity and SCI severity. These results indicate that the effects of the new FMT method on ASD severity also extend to the core symptoms of SCI and RRB (Figures 4A,B).

##### 3.2.1.2 SCI subscales

The SCI subscale includes four items: social consciousness (Awr), social cognition (Cog), social communication (Com), and social motivation (Mot). The severity reduction effects of the new FMT method on each item are shown in Supplementary Figure S1. The new FMT method reduced the severity of Awr, Cog, Com, and Mot scores by 24% (*p* < 0.001), 25% (*p* < 0.001), 27% (*p* < 0.001), and 36% (*p* < 0.001), respectively. The new FMT method had a slightly higher reduction in social motivation (Mot) compared to the overall reduction in SCI scores (28%), but other items showed similar reductions. These results indicate that the new FMT method's reduction in ASD severity applies almost uniformly to both SCI and RRB subcategories. Furthermore, the reduction in SCI severity also applies almost uniformly to the four subcategories.

#### 3.2.2 Gazefinder

In this study, we evaluated the effectiveness of the new FMT method for ASD using the SRS-2, which is aligned with the DSM-5 diagnostic criteria for ASD. However, because the SRS-2 is completed by parents and scored by examiners, the possibility of bias cannot be ruled out. Therefore, to confirm the reliability of the SRS-2 assessment, this study compared it with results from Gazefinder, which is expected to be an objective diagnostic aid for ASD.

In this clinical study, we used the pointing video (39) shown in Figure 1C to examine the correlation between SCI, a key component of the SRS-2 sociality scale, and Gazefinder results, which assess sociality.

Excluding three subjects who did not look at the geometric patterns or human figures at all, 27 subjects were included in the analysis.

As shown in the table below, the severity of SCI showed a statistically significant positive correlation with the amount of time spent looking at the geometric patterns and a statistically significant negative correlation with the amount of time spent looking at the human figures.

These results demonstrate that SCI, which accounts for a key component of the SRS-2, is highly reliable with minimal bias.

#### 3.2.3 SSP

Sensory processing disorder is one of the core symptoms of ASD, particularly restricted interests and repetitive behaviors. Approximately 90% of individuals with ASD have been reported to experience sensory processing disorders, including sensory hypersensitivity, sensory hyposensitivity, and hyporesponsiveness/sensation seeking (40, 41).

Sensory integration therapy, such as Ayres Sensory Integration Therapy (ASI), and environmental adjustments have been attempted to treat sensory processing disorders in children with ASD. Although favorable treatment outcomes have been reported for ASI, evidence supporting its therapeutic effectiveness remains limited (42). Furthermore, no previous attempts have been made to treat sensory processing disorders by intervening in the gut microbiota using methods such as prebiotics, probiotics, or FMT. Therefore, this clinical study marks the first attempt to treat sensory processing disorders using methods other than sensory integration therapy.

In this clinical study, the effects of a novel FMT method on sensory processing disorders were evaluated in 26 subjects diagnosed with sensory processing disorder by SSP. Changes in sensory hypersensitivity, sensory hyposensitivity, and hyporesponsiveness/sensation seeking were assessed before and after FMT.

**Figure 5:**
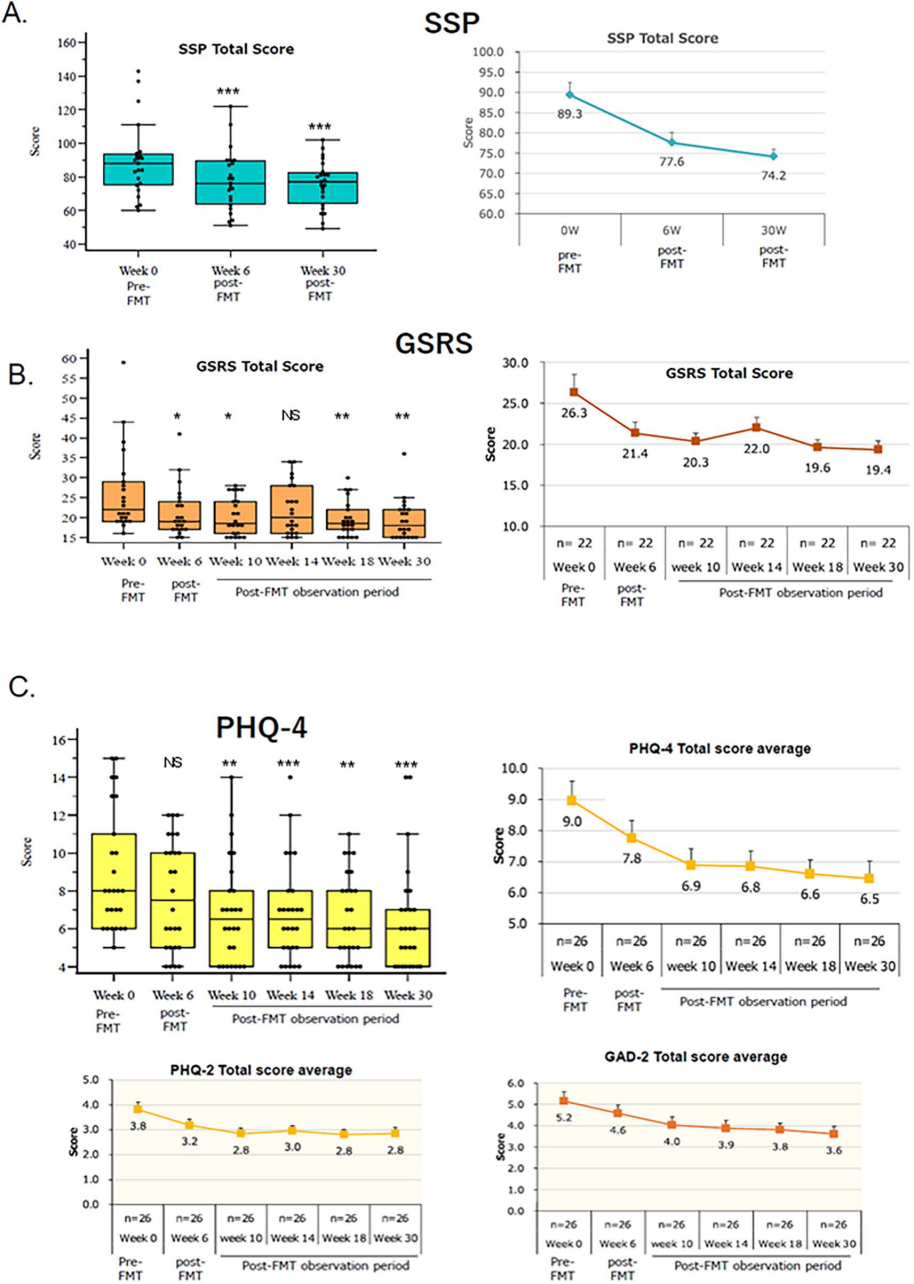
Effects of the novel FMT method on sensory processing disorder and ASD-related symptoms (gastrointestinal symptoms, anxiety, and depression). **(A)** Effect on reducing the severity of sensory processing disorder. (Left) Boxplot of SSP total score. Wilcoxon signed-rank test (Post-FMT vs Pre-FMT); ***p < 0.001. (Right) Changes in the mean SSP total score. Each data point represents the mean ± S.E.M. of *n* = 22. **(B)** Effect on reducing gastrointestinal symptoms. (Left) Boxplot of GSRS total score. Wilcoxon signed-rank test (Post-FMT vs Pre-FMT); *p < 0.05, **p < 0.01, NS, not significant. (Right) Changes in the mean GSRS total score. Each data point represents the mean ± S.E.M. of *n* = 22. **(C)** Effect on reducing anxiety and depression. (Upper, Left) Boxplot of PHQ-4 total score. Wilcoxon signed-rank test (Post-FMT vs Pre-FMT); ***p < 0.001, **p < 0.01, NS, not significant. (Upper, Right) Changes in the mean PHQ-4 total score. Each data point represents the mean ± SEM of *n* = 26. The PHQ-4 score is composed of the PHQ-2 (depression scale) and the GAD-2 (anxiety scale). (Lower, Left) Changes in the mean PHQ-2 total score. Each data point represents the mean ± SEM of *n* = 26. (Lower, Right) Changes in the mean GAD-2 total score. Each data point represents the mean ± SEM of *n* = 26.

As shown in Figure 5A, the new FMT method reduced the severity of sensory processing disorder by approximately 30% (*p* = 0.001), with the SSP score reaching a minimum of 38 points 30 weeks after the end of SHIN-1 administration.

Supplementary Figure S2 demonstrates the effectiveness of the new FMT method in reducing the severity of sensory hypersensitivity, sensory hyposensitivity, and hyporesponsiveness/sensation seeking. The new FMT method significantly reduced the severity of sensory hyposensitivity, sensory hypersensitivity, and hyporesponsiveness/sensation seeking by 23%, 28%, and 28%, respectively.

Based on these results, the reduction in sensory processing disorder by the new FMT method was approximately 30%, while the reduction in its subcomponents (sensory hypersensitivity, sensory hyposensitivity, and hyporesponsiveness/sensation seeking) ranged from 23% to 28%. These effects were not significantly different from the reduction observed in ASD severity (29%).

#### 3.2.4 GSRS and BSFS

Many studies have reported that 30%–70% of patients with ASD experience gastrointestinal disorders (43, 44). Among these, abdominal pain (59%) is the most common, followed by constipation (51%) and diarrhea (43%) (43, 44).

Therapies that target the gut microbiota, such as antibiotics (16), prebiotics, probiotics, and FMT, have been investigated (45, 46), resulting in improvements in behavior and symptoms (47, 48). In the field of FMT, Kang et al. demonstrated favorable results for gastrointestinal disorders using FMT in combination with antibiotics (17).

In this study, the effect of a new FMT method on the severity of gastrointestinal disorders was evaluated using the GSRS and BSFS. The GSRS assessed 22 of the 30 patients who had gastrointestinal disorders before FMT. The BSFS, on the other hand, assessed all subjects.

Figure 5B shows the effect of a new FMT method on gastrointestinal disorders. The new FMT method reduced the severity of gastrointestinal disorders by approximately 61% (*p* = 0.001), with a lower limit of 15 points. The effect size significantly exceeded the reduction in ASD severity reported above. The mean score decreased to 19.4, approaching the baseline value of 15, indicating normalized outcomes in many participants.

The BSFS is a widely used tool for evaluating the therapeutic effects of various intestinal disorders. It classifies and evaluates stool on a seven-point scale, with 1 and 2 representing hard stools associated with constipation, 3 and 4 representing normal stool, and 5 through 7 representing a range from soft stools to diarrhea. Before FMT, 60% of study subjects had normal stools of types 3 and 4. After FMT, the proportion of subjects with ideal stools of type 4 gradually increased, reaching 87% after 30 weeks. These results indicate that of 12 subjects whose stools were outside the normal range, approximately 8 patients achieved a normal range, representing a 67% improvement rate. This value is similar to the improvement rate of gastrointestinal disorders (61%), supporting the effectiveness of the new FMT for gastrointestinal disorders.

#### 3.2.5 PHQ-4 (PHQ-2 and GAD-2)

A large-scale meta-study by Lai et al. found that 42% to 56% of patients with ASD experience anxiety symptoms, and 12% to 70% experience depressive symptoms (49, 50).

In this study, we evaluated the effectiveness of the new FMT method in reducing the severity of depression and anxiety symptoms in 26 patients (86.7% of the total) with depression and anxiety symptoms using the PHQ-4 (PHQ-2 and GAD-2). The results are shown in Figure 5C. The new FMT method reduced the severity of anxiety and depressive symptoms by approximately 50% (*p* < 0.001). This result significantly exceeded the reduction in ASD severity observed using the SRS-2 (approximately 29%).

### 3.3 Adverse events of the new FMT

In this clinical study, no adverse events, even mild ones, were observed among the 30 subjects.

Meanwhile, in the prior ASD-FMT studies (17, 18, 30), no serious adverse events were observed, with only mild adverse events reported in a few cases.

Recently, surveys of adverse events with conventional FMT methods have reported an overall incidence of 24% (1,347/5,688), including serious events in 6% (246/4,241) of patients (51, 52).

Although the patient number in this trial was small, these results suggest that the new FMT method has a lower incidence of adverse events. Future studies with larger patient cohorts will further confirm its safety.

## 4 Discussion

This study evaluated the effectiveness of a new FMT method for ASD using a pre- and post-trial comparative study rather than a randomized controlled trial. There are two reasons for this. (1) FMT for ASD is a new treatment, and to confirm its efficacy and safety, an exploratory study that allows detailed observation of changes before and after treatment is considered appropriate. (2) When targeting children with ASD, a randomized controlled trial that randomly assigns patients to a treatment group or a placebo group poses high ethical hurdles, and there is also the possibility of resistance to being assigned to a placebo group when a potential treatment is expected. Therefore, a pre- and post-trial comparative study was selected, in which all patients are first treated and changes observed.

### 4.1 SRS-2

The SRS-2 used in this study is a test completed by parents and scored by an examiner, so the possibility of bias cannot be completely eliminated. Therefore, its diagnostic performance has been extensively verified in large-scale studies involving a large number of children with ASD and typically developing children. The diagnostic performance of the SRS-2 (or SRS) was evaluated using indices derived from receiver operating characteristic (ROC) curves, including area under the curve (AUC), sensitivity, specificity, positive predictive value, and negative predictive value. These values have consistently been shown to be in the range of approximately 80% to over 90% (22, 23). Furthermore, when the diagnostic performance of the SRS-2 (and SRS) was compared with that of the ADOS and ADI-R, it was demonstrated that the SRS-2 exhibits good inter-rater reliability, high internal consistency, and convergent validity with Autism Diagnostic Observation Schedule (ADOS), Autism Diagnostic Interview-Revised (ADI-R), and the Social Communication Questionnaire (SCQ) (23, 24, 53). Therefore, the SRS-2 is now considered an objective assessment method. Furthermore, to further confirm the lack of bias in the SRS-2 results despite being answered by parents, the results were objectively verified using the gaze detection device Gazefinder, which has been confirmed to correlate with the SRS-2 social skills index.

### 4.2 Effect of the new FMT method on core symptoms of ASD

In this study, a new FMT method using fecal microbial solution SHIN-1 containing hydrogen nanobubble water was administered to a total of 30 subjects: 28 with a severe SRS-2 rating, 1 with a moderate rating, and 1 with a normal rating.

As a result, the subjects' SRS-2 severity scores had already significantly improved six weeks after FMT, and 30 weeks later, the subjects' severity scores had statistically significantly decreased by approximately 30%. When this reduction was analyzed as a change in the number of subjects by severity, 19 of the 20 subjects (28 with a severe rating and 1 with a moderate rating) transitioned to the mild range, including the normal range, demonstrating a significant effect.

In contrast, previous studies (17, 18, 30, 54) on the therapeutic effects of FMT for ASD have limited their subjects to children with ASD who also had gastrointestinal disorders.

In this clinical study, 22 of the 30 subjects had gastrointestinal disorders, while 8 did not. The effects of the new FMT method varied significantly between these groups. Specifically, the new FMT method achieved a 24% reduction in symptoms in children with ASD who had gastrointestinal disorders, compared with a 45% reduction in symptoms in children without gastrointestinal symptoms. In the latter group, the mean SRS-2 scores of children with ASD after FMT were within the normal range.

Many previous studies examining the relationship between ASD symptoms and gastrointestinal disorders have concluded that gastrointestinal disorders are not associated with the severity of ASD (55, 56). However, some reports suggest a correlation, and no clear conclusions have been reached at this time (57). The results of this clinical study, demonstrating significant differences in FMT outcomes depending on the presence or absence of gastrointestinal disorders, represent an interesting new finding. Future studies will involve a larger sample size, including the subgroup of children with ASD who do not have gastrointestinal disorders, and will explore methods to further improve the effectiveness of FMT for children with ASD who do have gastrointestinal disorders.

Furthermore, this new FMT method demonstrated a 30% reduction in each of the core symptoms of ASD, including SCI and RRB. This effect was also observed in the four subscales of SCI (social awareness, social cognition, social communication, and social motivation) and sensory processing disorder included in RRB, and the results were similar to the reduction effect on ASD severity described above.

Regarding sensory processing disorder, a recent study by Watanabe et al. (58) suggests a common neural basis between sensory symptoms, such as restricted interests, a core symptom of ASD, and higher-level cognitive symptoms. This may explain the similar reduction effects of the new FMT method on both RRB (restricted interests and repetitive behaviors) and sensory processing disorder symptoms.

Interestingly, the effects of this new FMT method also extended to gastrointestinal disorders, depression, and anxiety symptoms commonly seen in children with autism spectrum disorder (ASD). The reduction rates were 61%, 56%, and 50%, respectively, approximately twice the effect observed for core ASD symptoms.

Thus, this new FMT method demonstrates the high efficacy of FMT alone in treating ASD symptoms. Furthermore, the scope and magnitude of the effects extend beyond the core symptoms of ASD, which are thought to have different neural substrates, and their subcategories, such as sensory processing disorder, to gastrointestinal disorders, depression, and anxiety. This demonstrates the broad and comprehensive impact of this new FMT method.

These results demonstrate the potential for FMT intervention in the gut microbiota to broadly affect brain function via the gut microbiota-gut-brain axis. It is also noteworthy that no adverse events were observed in this study. On the other hand, previous studies reported no severe adverse events, with only two or three minor ones documented (17.18) and recent surveys of adverse events associated with existing FMT methods have shown that adverse events, including serious ones, occurred in 26% of patients (51, 52). Compared with these results, this new FMT method suggests its exceptional safety.

Furthermore, the results of this clinical study demonstrate that this new FMT method is a highly safe methodology that effectively enables “colonization, reconstitution, and maintenance” without the need for concomitant antibiotics, by administering extremely small amounts of microbiota solution alone.

Thus, the novel FMT method may represent a safer and more effective alternative to conventional approaches, although further validation is required (18, 30).

As mentioned above, the novel FMT method, unlike previous methods, is a methodology that maintains its effectiveness without the use of antibiotics. However, the underlying mechanism remains unclear at present. However, recent studies have shown that hydrogen-rich water (HRW) suppresses intestinal inflammation, thereby maintaining the integrity of the intestinal barrier and activating butyrate-producing bacteria (59–63). Specifically, it has already been reported that microbial solutions prepared under anaerobic conditions promote the colonization and proliferation of obligate anaerobic bacteria and inhibit the growth of pathogenic bacteria (64). Furthermore, hydrogen nanobubble water is known to have strong reducing activity and effective scavenging properties against reactive oxygen and nitrogen species (65). Based on these properties, we hypothesized that the use of hydrogen nanobubble water could simultaneously promote the colonization and proliferation of obligate anaerobic bacteria in the transplanted microbial solution and inhibit the growth of pathogenic bacteria. Our research results suggest that FMT using a microbial solution prepared with hydrogen nanobubble water under anaerobic conditions leads to the production of short-chain fatty acids, a decrease in intestinal pH, an increase in beneficial bacteria such as Bacteroides, and the inhibition of the growth of pathogenic bacteria. These changes may improve neurotransmitter imbalances in the brain and contribute to a reduction in core symptoms of ASD, supporting the hypothesis that FMT using hydrogen nanobubble water has beneficial effects on the composition of the gut microbiota and host physiology.

Furthermore, this clinical study was conducted under strict quality control of SHIN-1 in accordance with pharmaceutical GMP standards.

To maintain the bacterial composition of SHIN-1 within a consistent range—one element of SHIN-1 quality—and ensure the reproducibility of SHIN-1 transplant results, this clinical study used SHIN-1 prepared from the feces of the same donor.

A 30-week clinical study was conducted using SHIN-1 with the quality guaranteed above, and no adverse event was observed among the 30 study subjects.

To widely disseminate this new FMT method, it is necessary to establish a method for preparing donor bacterial solutions with a consistent bacterial composition ratio using stool from multiple donors. We are currently investigating the preparation of such donor bacteria and are considering conducting clinical trials using this new FMT method.

## 5 Limitations

Several limitations should be acknowledged. First, this was a pre–post study without a randomized control group. Placebo effects, regression to the mean, or natural developmental trajectories cannot be completely excluded, although the magnitude and persistence of improvements, together with objective eye-tracking and microbiome data, argue against a purely nonspecific explanation. Second, the sample size was modest (*N* = 30), limiting statistical power for subgroup analyses (e.g., GI vs. non-GI, age, sex). Third, a single donor was used; while this improves standardization, it also limits generalizability and precludes evaluation of donor-specific effects.

Fourth, although we assessed multiple clinical domains, we did not include direct cognitive or academic performance measures, which may be relevant for understanding functional outcomes. Fifth, the microbiome analyses, while demonstrating global reconstruction, did not yet define a precise microbial “signature” of response; further metagenomic and metabolomic profiling is warranted.

Finally, all participants were recruited in Japan, and cultural or healthcare-system differences may influence generalizability to other populations. Nonetheless, the biological mechanisms of the gut–brain axis are likely to be broadly similar across contexts.

## 6 Future directions

Future research should prioritize:

- **Randomized, placebo-controlled trials** comparing SHIN-1 with standard care and/or conventional FMT.
- **Dose–response and timing studies** to determine optimal schedules, booster doses, and the durability of effects beyond one year.
- **Stratified analyses** based on GI phenotype, baseline microbiome composition, and clinical profile to identify predictors of response.
- **Mechanistic studies** integrating metagenomics, metabolomics (e.g., SCFAs, bile acids), immune markers, and neuroimaging to clarify pathways linking microbiota changes to brain function.
- Development of a more effective FMT method through additional standalone administration of hydrogen nanobubble water before and during SHIN-1 administration, based on elucidating the primary role of hydrogen nanobubbles in SHIN-1.
- **Longitudinal developmental follow-up**, including cognitive, adaptive, and educational outcomes, to assess whether early microbiota-targeted interventions can alter long-term trajectories in ASD.

If replicated and extended, SHIN-1 and related hydrogen nanobubble–based FMT technologies may establish a new class of microbiota therapeutics for neurodevelopmental disorders.

## 7 Conclusion

This new FMT method using a fecal microbiota solution (SHIN-1) containing hydrogen nanobubble water alleviates core and peripheral symptoms of ASD. It does not require antibiotics or intestinal cleansing, and by transplanting a smaller amount of bacteria than conventional FMT, it has the potential to be a groundbreaking treatment that is sustainable, safe, and less burdensome for patients. Based on the new findings obtained in this clinical trial, we hope to conduct a double-blind clinical trial in the future.

## Data Availability

The datasets presented in this study can be found in online repositories. The names of the repository/repositories and accession number(s) can be found below: Trial ID: jRCTs031230041 https://jrct.mhlw.go.jp/en-latest-detail/jRCTs031230041.

## Supplementary material

Supplementary Figure S1 The severity results for the SCI subscales social awareness (Awr), social cognition (Cog), social communication (Com), and social trembling (Mot) are shown in Supplementary Figure S1.

Supplementary Figure S2 The results for the severity of the sensory processing disorder subscales hypersensitivity, hyposensitivity, and sensory response/sensation seeking are shown in Supplementary Figure S2.
