# Supplementary figures and images for: Safety and efficacy of a novel fecal microbiota transplantation method using hydrogen nanobubble water without antibiotics or bowel cleansing in children with autism spectrum disorder: an open-label, single-arm study demonstrating improvements in core and comorbidity symptoms
Source: Front Pediatr. 2026 Mar 11;14:1767346. doi: 10.3389/fped.2026.1767346 (PMC13013415; doi:10.3389/fped.2026.1767346)

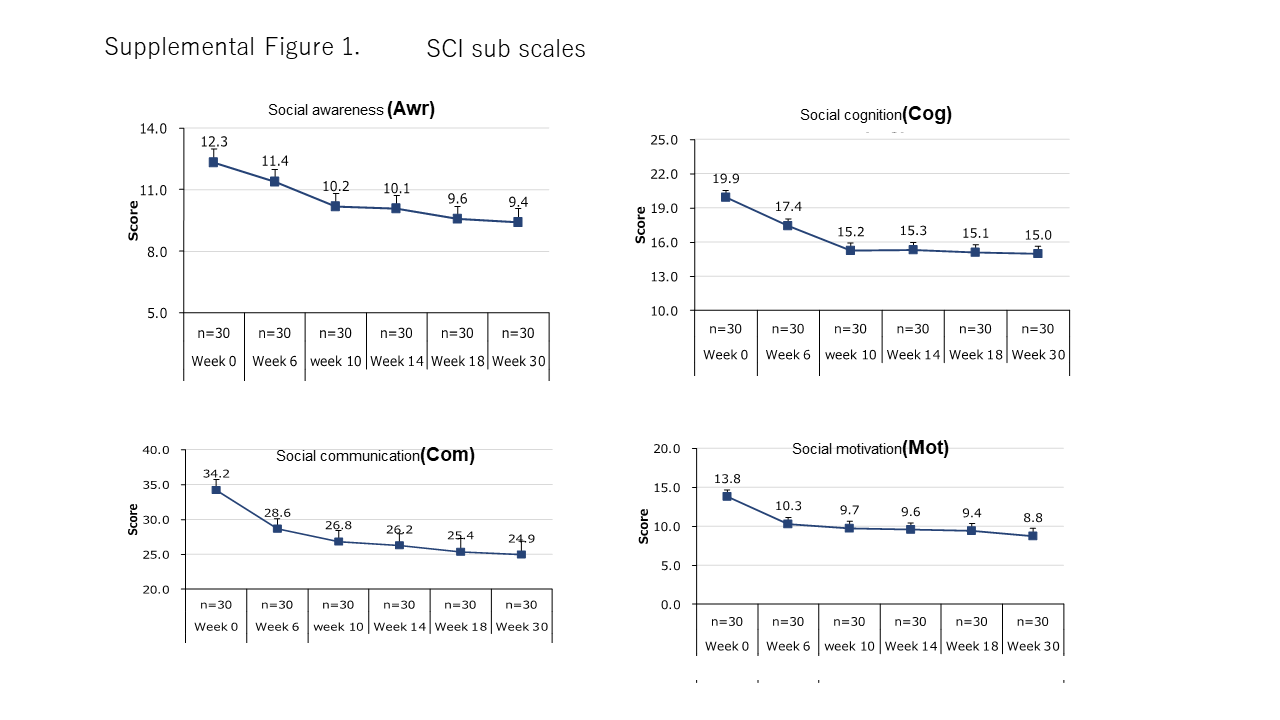

Supplement: Supplementary Figure S1 — The severity results for the SCI subscales social awareness (Awr), social cognition (Cog), social communication (Com), and social trembling (Mot) are shown in Supplementary Figure S1. [file Image1.tif]

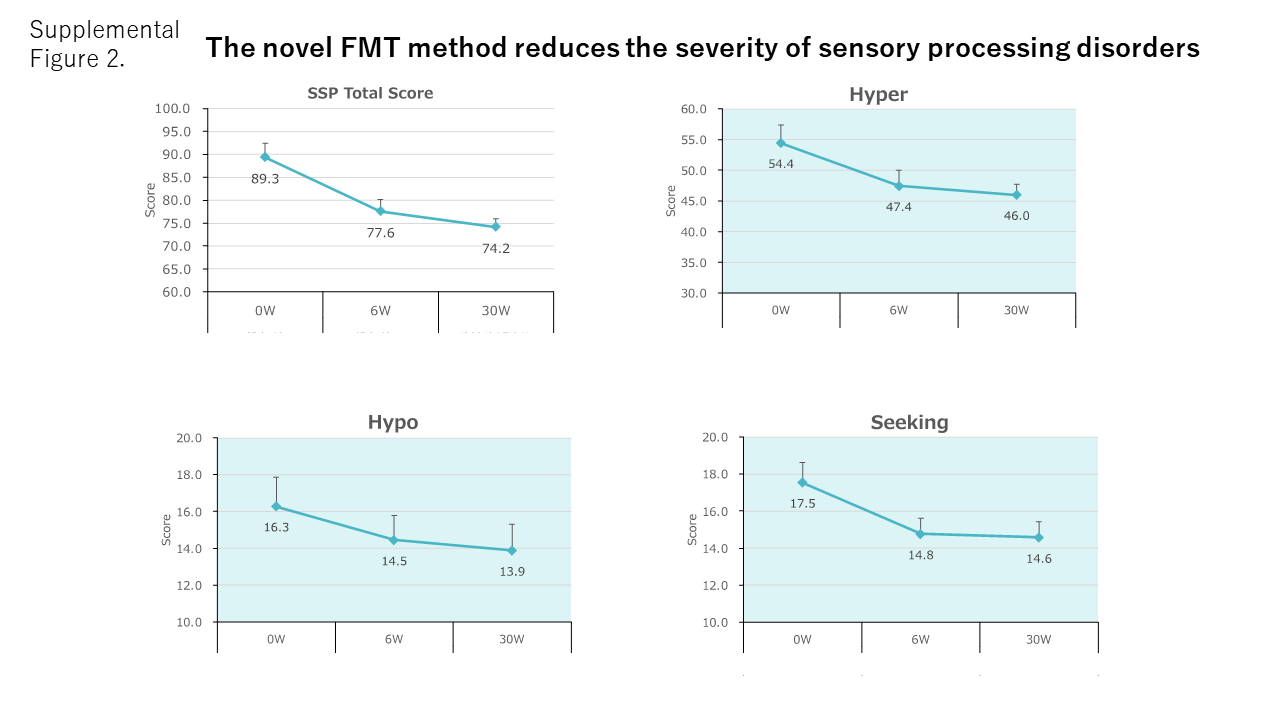

Supplement: Supplementary Figure S2 — The results for the severity of the sensory processing disorder subscales hypersensitivity, hyposensitivity, and sensory response/sensation seeking are shown in Supplementary Figure S2. [file Image2.tif]
